# Comparative Analysis of Corneal Higher-Order Aberrations after Laser-Assisted In Situ Keratomileusis, Photorefractive Keratectomy, and Small Incision Lenticule Extraction with Correlations to Change in Myopic Q-Value and Spherical Equivalent with and without Astigmatism

**DOI:** 10.3390/jcm13071906

**Published:** 2024-03-26

**Authors:** Majid Moshirfar, Soroush Omidvarnia, Michael T. Christensen, Kaiden B. Porter, Josh S. Theis, Nathan M. Olson, Isabella M. Stoakes, Carter J. Payne, Phillip C. Hoopes

**Affiliations:** 1 Hoopes Vision Research Center, Hoopes Vision, 11820 S. State St., Ste. 200, Draper, UT 84020, USA; isabellamstoakes@gmail.com (I.M.S.); cjp127@case.edu (C.J.P.); pch@hoopesvision.com (P.C.H.); 2 John A. Moran Eye Center, University of Utah School of Medicine, Salt Lake City, UT 84132, USA; 3 Utah Lions Eye Bank, Murray, UT 84107, USA; 4 School of Medicine, Texas Tech University, Lubbock, TX 79409, USA; somidvar@ttuhsc.edu; 5 School of Medicine, Wake Forest University, Winston Salem, NC 27103, USA; michael.christensen218@gmail.com; 6 School of Medicine, University of Arizona, Phoenix, AZ 85004, USA; kaidenporter@arizona.edu (K.B.P.); joshstheis@arizona.edu (J.S.T.); 7 College of Osteopathic Medicine, Rocky Vista University, Ivins, UT 84738, USA; nathan.olson@ut.rvu.edu; 8 School of Osteopathic Medicine, Pacific Northwest University of Health Sciences, Yakima, WA 98901, USA; 9 School of Medicine, Case Western Reserve University, Cleveland, OH 44106, USA

**Keywords:** higher-order aberrations, vertical coma, horizontal coma, trefoil, spherical aberrations, LASIK, SMILE, PRK, refractive surgery, myopia, cornea, spherical equivalent, Q-value, asphericity, astigmatism

## Abstract

**Background**: This retrospective chart review compared the higher-order aberrations (HOAs) among photorefractive keratectomy (PRK), laser-assisted in situ keratomileusis (LASIK), and small incision lenticule extraction (SMILE) alongside changes in spherical equivalent (SEQ) and corneal shape (Q-value). **Methods**: Analyzing 371 myopic eyes, including 154 LASIK, 173 PRK, and 44 SMILE cases, Pentacam imaging was utilized pre-operatively and at one-year post-operative visits. **Results**: All procedures resulted in 100% of patients achieving an uncorrected distance visual acuity (UDVA) of 20/40 or better, with 87% of LASIK and PRK, and 91% of SMILE patients having 20/20 or better. Significant increases in HOAs were observed across all procedures (*p* < 0.05), correlating positively with SEQ and Q-value changes (LASIK (0.686, *p* < 0.05), followed by PRK (0.4503, *p* < 0.05), and SMILE (0.386, *p* < 0.05)). Vertical coma and spherical aberration (SA) were the primary factors for heightened aberration magnitude among the procedures (*p* < 0.05), with the largest contribution in SMILE, which is likely attributed to the centration at the corneal apex. Notably, PRK showed insignificant changes in vertical coma (−0.197 µm ± 0.0168 to −0.192 µm ± 0.0198, *p* = 0.78), with an increase in oblique trefoil (*p* < 0.05). **Conclusions**: These findings underscore differences in HOAs among PRK, LASIK, and SMILE, helping to guide clinicians.

## 1. Introduction

By 2050, it is estimated that nearly 4.8 billion individuals, equivalent to approximately 50% of the world’s population, will be affected by myopia [1]. In response to this growing challenge, the ophthalmic field has witnessed transformative advancements with the introduction and evolution of procedures like photorefractive keratectomy (PRK), laser-assisted in situ keratomileusis (LASIK), small incision lenticule extraction (SMILE) [2].

These procedures have evolved significantly since their respective inceptions, with advancements in technology and surgical techniques enhancing patient outcomes. While it is recognized that these surgeries can induce higher-order aberrations (HOAs) [3], which manifest as glare, halos, and reduced contrast sensitivity, especially in low light, the field has made considerable strides in addressing these challenges. Innovations like wavefront-optimized and topography-guided ablations have significantly reduced the induction of corneal HOAs [4], reflecting the ongoing progress in this area.

While individual studies have made efforts to compare the induction of HOAs between two surgical methods, comprehensive research that compares PRK, LASIK, and SMILE remains sparse. This study aimed to fill this gap by analyzing the differences in corneal HOA induction across these procedures and examining the interrelations between HOA induction, alterations in the spherical equivalent (SEQ), and changes in corneal asphericity.

## 2. Materials and Methods

### 2.1. Study Design

Data examined in this non-randomized retrospective chart review were from a single tertiary center in Draper, Utah. The procedures were performed by a single surgeon (M.M.). The study was approved by the Hoopes Vision Ethics Committee, which adheres to the tenets of the Declaration of Helsinki. This study was also approved by the Biomedical Research Alliance of New York (BRANY, Lake Success, NY, USA) Institutional Review Board (#20-12-547-823) on 13 November 2020.

### 2.2. Inclusion and Exclusion Criteria

Patients included in the study were those who underwent myopic corrections of −1.25 to −9.25 D via LASIK, PRK, or SMILE in one or both eyes from 14 March 2017 to 15 February 2022. Of the 371 eyes (200 patients) involved in this study, 154 eyes (83 patients) underwent LASIK, 173 (93 patients) underwent PRK, and 44 eyes (24 patients) received SMILE. Patients with a corneal pachymetry of <500 µm received PRK. It is noteworthy that patients with a pachymetry in the range of 500–550 µm could have been directed towards PRK if they required a correction of 6.0 D or more. Only patients who had a pre-operative and one-year post-operative Pentacam (HR; Oculus, Wetzlar, Germany) assessment with a measurement status of “OK” were included. Patients presenting with specific ocular conditions such as age-related macular degeneration, keratoconus, prior retinal tear, emerging cataracts, glaucoma, or other pathologies that might influence the outcomes were excluded. Those in need of laser enhancement were also excluded.

### 2.3. Surgical Methods

LASIK: Flaps were created to be 8.7–9 mm in diameter and 100 µm thick. They were completed using the FS200 (Alcon Laboratories, Inc., Fort Worth, TX, USA) femtosecond laser. The WaveLight EX500 Excimer Laser System (Alcon Laboratories, Inc., Fort Worth, TX, USA) was the excimer laser used for corneal ablation. Ablation zones were 6.5 mm with a transition zone of up to 9.0 mm. Post-operative treatment included topical ofloxacin or moxifloxacin (0.3 or 0.5%, respectively) and prednisolone acetate 1%. The steroid was to be used every hour for the remainder of the day following the procedure. The steroid and antibiotic were then used q.i.d. for 1 week.

PRK: The corneal epithelium was removed after 20 s application of 18% alcohol. This was followed by ablation using WaveLight EX500 Excimer Laser System (Alcon Laboratories, Inc., Fort Worth, TX, USA) with an ablation a zone of 6.5 mm central ablation and up to a 9 mm transitional zone. If the ablation depth went beyond 65 µm, then Mitomycin C 0.02% was given for approximately 20 s. Post-operatively, an Acuvue Oasys (Johnson & Johnson Vision Care, Inc., Jacksonville, FL, USA) bandage contact was applied for five to seven days, until epithelialization was complete. Topical moxifloxacin 0.5% was prescribed for four drops daily for a total of seven days, and prednisolone acetate was prescribed at four drops daily for one month. After one month, fluorometholone 0.1% drops were applied three times per day for three weeks and then tapered: b.i.d. for another three weeks and then q.d. for three weeks.

SMILE: The VisuMax 500 kHz femtosecond laser (Carl Zeiss Meditec, Jena, Germany) was used to create the lenticule, which was preset to be either 6.0 mm with a 0.5 mm transition zone for toric treatments or 6.5 mm for spherical treatment. This was completed by using a laser energy of 125 nJ, with cap thickness and diameter inputted at 120 µm and 7.5 mm, respectively. A 3.5 mm superior incision was used at a 90-degree meridian with a side-cut angle of 90 degrees. The measurements for lenticular spot separation, side cut, cap cut, and cap angle were 3.7 µm, 2.0 µm, 3.8 µm, and 2.0 µm, respectively. Post-operative drops included topical ofloxacin 0.3% or moxifloxacin 0.5% and prednisolone acetate 1%. The antibiotic was dosed q.i.d. for 1 week, and the steroid was tapered: q.i.d. for one week, b.i.d. for one week, and q.d. for two weeks.

### 2.4. Study Measurements

Measurements included root-mean-square (RMS) values for the full thickness of the cornea. The primary outcome variables were visual metrics one year post-surgery, including uncorrected distance visual acuity (UDVA), corneal HOAs, manifest refraction, changes in corrected distance visual acuity (CDVA), SEQ, and safety/efficacy indices. The HOAs analyzed, which were characterized by their respective Zernicke coefficients, included horizontal trefoil (Z3,3), horizontal coma (Z3,1), vertical coma (Z3,−1), oblique trefoil (Z3,−3), and spherical aberration (SA) (Z04,4), which were based on a 6 mm optical zone. Differences between pre-operative and post-operative HOA values were analyzed for statistical significance. Additionally, ratios of change in HOAs to change in SEQ (ΔHOA/ΔSEQ), along with changes in Q-value (ΔQ), and SA (ΔSA) were measured and examined.

### 2.5. Statistical Methods and Data Analysis

The statistical analysis, along with the generation of graphs and tables, were conducted using Microsoft Excel 2016 (Microsoft Inc., Redmond, VA, USA) and IBM SPSS Statistics for Windows, Version 29.0 (IBM Corp., Armonk, NY, USA). Basic descriptive statistical computations, including means and standard deviations were carried out to summarize the data. The Shapiro–Wilk test was employed to assess the normality of data distributions, guiding the choice between parametric and non-parametric tests. For quantitative demographic data that conformed to normal distribution and homogeneity of variances, one-way ANOVA and independent samples t-tests were utilized for comparisons across the three surgical procedures. Qualitative or categorical data were analyzed using Chi-squared tests to evaluate the distribution of frequencies across different categories.

In instances where data did not meet the assumptions of normality, non-parametric tests were applied. The Wilcoxon rank-sum test was utilized to compare median values of non-normally distributed variables between two independent groups. For multiple pairwise comparisons, the Bonferroni adjustment was employed to correct for the increased risk of Type I errors.

Pre-operative data, post-operative data, and their associated delta values were assessed through generalized estimating equations (GEEs) to account for inter-eye variability. Statistical significance was determined to be *p* < 0.05.

## 3. Results

### 3.1. Pre-Operative Characteristics

The levels of myopic sphere and SEQ correction were greater for SMILE patients, being recorded at −5.185 D ± 0.350 and −5.406 D ± 0.346, respectively (both *p* < 0.001), while a reduced pre-operative cylinder was observed, at −0.443 D ± 0.072 (*p* = 0.011) compared to PRK and LASIK.

Pachymetry measurements (minimum, apex, and pupil pachymetry) showed thinner results at 522.664 µm ± 3.217, 526.850 µm ± 3.179, and 526.26 µm ± 3.185, respectively, in the PRK group when compared to the LASIK/SMILE groups (all *p* < 0.001). Furthermore, the PRK cohort displayed elevated total HOAs (0.43 µm ± 0.012) and vertical coma (−0.197 µm ± 0.021) relative to the other procedures (both *p* < 0.001). Patient demographics can be found in Table 1. 

### 3.2. Visual Outcomes

**Figure A1:**
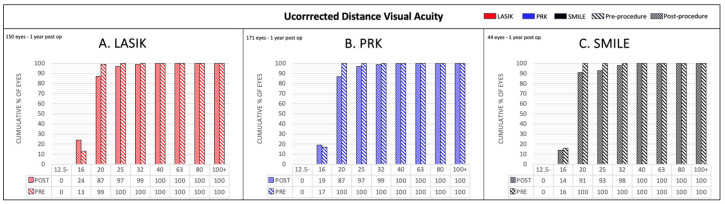
One-year post-operative uncorrected distance visual acuity (UDVA) compared to pre-operative corrected distance visual acuity (CDVA) by surgery type (laser-assisted in situ keratomileusis, LASIK ((**A**); red); photorefractive keratectomy, PRK ((**B**); blue); small incision lenticule extraction, SMILE ((**C**); black)).

**Figure A3:**
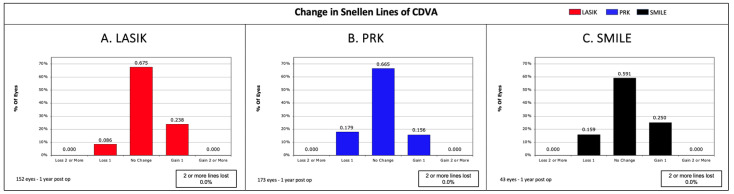
One-year post-operative changes in Snellen lines of CDVA by surgery type (LASIK ((**A**); red), PRK ((**B**); blue), and SMILE ((**C**); black)).

Across all procedures, 100% of patients achieved a UDVA of 20/40 or better. Specifically, 87% of LASIK and PRK patients, along with 91% of SMILE patients, attained a UDVA of 20/20 or better (Figure A1). CDVA analysis revealed a loss of one Snellen line in 9% of LASIK, 22% of PRK, and 16% of SMILE patients. Conversely, 24% of LASIK, 16% of PRK, and 25% of SMILE patients experienced an improvement of one Snellen line in CDVA. Notably, no patient across all procedures had a change of two or more Snellen lines in CDVA (Figure A3).

Safety indices (post-operative BCVA/pre-operative BCVA) for LASIK, PRK, and SMILE were 1.027, 1.003, and 1.031, respectively. Efficacy indices (post-operative UCVA/pre-operative BCVA) were recorded at 0.993 for LASIK, 0.974 for PRK, and 0.968 for SMILE. Regarding procedure accuracy, 93% of LASIK, 95% of PRK, and 86% of SMILE patients were within 0.5 D of the intended SEQ. When the range was extended to 1.0 D, these figures jumped to 99% for LASIK, 100% for PRK, and 100% for SMILE.

### 3.3. Higher-Order Aberration (HOA) Analysis

All three refractive procedures showed significant increases in both HOAs and spherical aberration (SAs, Z0,4) post-operatively (*p*-value < 0.05). SMILE demonstrated the largest change in these aberrations, where total HOAs and spherical aberration increased from 0.329 and 0.183 µm pre-operatively to 0.617 and 0.305 µm post-operatively, respectively. Vertical coma (Z3,−1) increased notably in LASIK (from −0.0922 µm to −0.127 µm) and SMILE (from −0.0989 µm to −0.323 µm) patients, while oblique trefoil (Z3,−3) only showed a significant reduction in PRK patients (from −0.010 µm to −0.041 µm). In contrast, horizontal coma (Z3,1) and horizontal trefoil (Z3,3) did not exhibit significant alterations post-procedure across all methods (Table 2).

**Figure 1:**
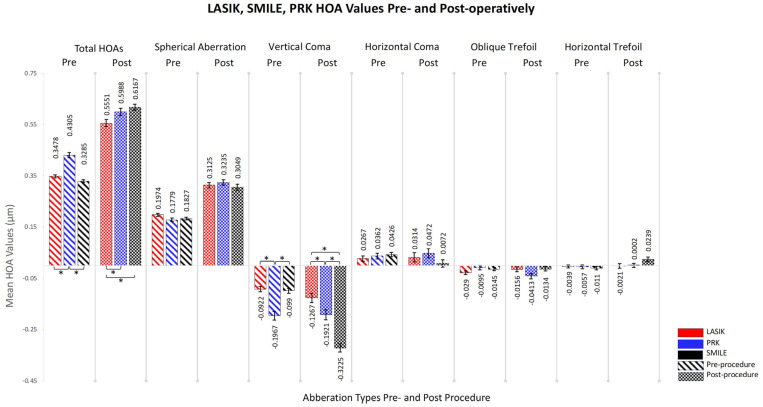
**Visualization of cumulative HOA induction for each surgical procedure**. The colored bars differentiate the surgical procedures: LASIK is in red, PRK is in gray, and SMILE is in black. Each aberration type has its own column, showcasing pre-operative values on the left and post-operative values on the right for each procedure. Error bars signify the standard error of the measurements. PRK = photorefractive keratectomy, LASIK = laser-assisted in situ keratomileusis, SMILE = small incision lenticule extraction, HOAs *=* higher-order aberrations. Statistical significance is indicated by an asterisk (*) above the bars, with *p* < 0.05.

Pairwise comparisons indicated significant pre-test differences between SMILE and PRK and between PRK and LASIK for total HOAs (*p*-value < 0.05). Post-test, LASIK differed significantly from both PRK and SMILE. Similarly, vertical coma showed significant pre-test differences in LASIK vs. PRK and PRK vs. SMILE, with all post-test comparisons (LASIK vs. PRK, LASIK vs. SMILE, PRK vs. SMILE) also revealing significant differences (*p*-values < 0.05) (Figure 1).

There were statistically significant differences in the median changes in total HOAs, spherical aberration, and vertical coma across all procedures (*p* < 0.05). Significant differences in total HOAs and vertical coma changes were identified post-SMILE compared to both LASIK and PRK (*p* < 0.05). Additionally, significant changes in spherical aberration and oblique trefoil were noted between LASIK and PRK (*p* < 0.05).

**Figure 2:**
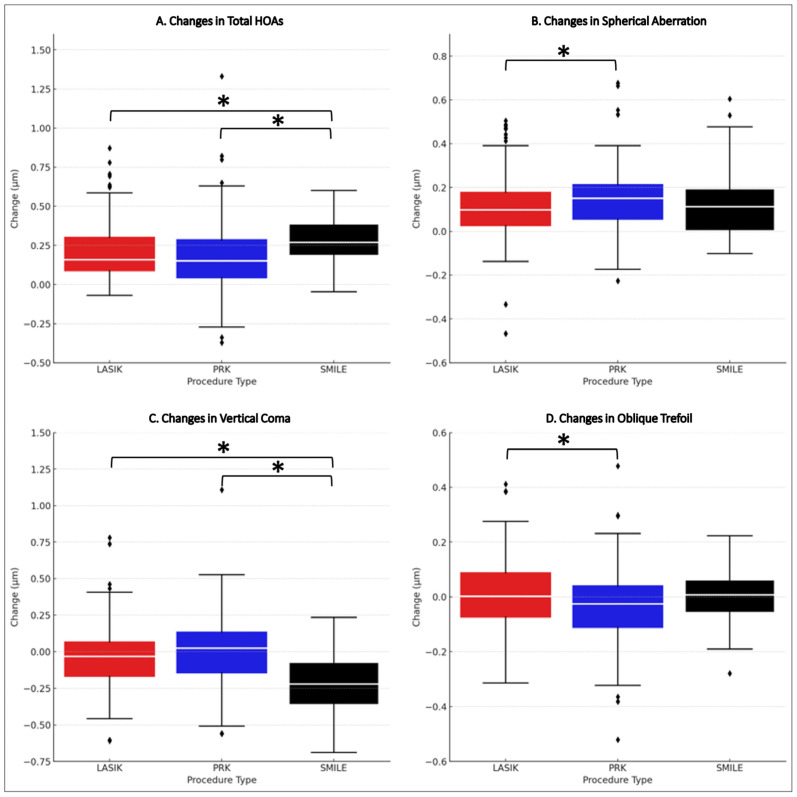
**Differential HOA progression patterns post-refractive procedures**. A composite analysis of total HOAs (**A**), spherical aberration (**B**), vertical coma (**C**), and oblique trefoil (**D**) showcases the variability in vision damage changes after LASIK, PRK, and SMILE surgeries. Medians are emphasized with white lines across the colored boxes—red for LASIK, blue for PRK, and black for SMILE. Asterisk (*) = statistical significance, Diamonds = individual data points that are beyond the whiskers of the box plots, PRK = photorefractive keratectomy, LASIK = laser-assisted in situ keratomileusis, SMILE = small incision lenticule extraction, HOAs = higher-order aberrations.

SMILE (0.2880 µm ± 0.1607) resulted in a median increase in total HOAs that was greater than those of both LASIK (0.2070 µm ± 0.1974, effect size: 0.335) and PRK (0.1680 µm ± 1.3092, effect size: 0.410). For spherical aberration, PRK showed a median increase of 0.1460 µm ± 0.1666 compared to that of LASIK (0.1160 µm ± 0.1665, effect size: 0.160). The median change in vertical coma with SMILE (0.2240 µm ± 0.2403) was greater than both LASIK (effect size: −0.4786) and PRK (effect size: −0.559). Moreover, the median change in oblique trefoil with PRK (−0.0318 µm ± 0.1802) was greater than with LASIK (effect size: −0.194) (Figure 2).

### 3.4. Correlation Analysis

**Figure 3:**
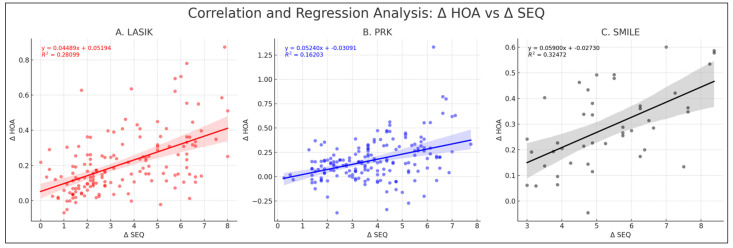
**Correlation and regression analysis of Δ HOAs vs. Δ SEQ.** This figure consists of three plots representing the correlation and regression analysis for the change in Δ HOAs against the change in Δ SEQ across LASIK (**A**), PRK (**B**), and SMILE (**C**). Each scatter plot with a linear regression line shows the equation of the regression line and the corresponding R^2^ value. PRK = photorefractive keratectomy, LASIK = laser-assisted in situ keratomileusis, SMILE = small incision lenticule extraction, HOAs = higher-order aberrations, SEQ = spherical equivalent.

Analysis indicated non-normal distribution for Δ SEQ and Δ total HOAs in LASIK and PRK, with *p*-values of <0.05 for LASIK and PRK. In contrast, for SMILE, Δ SEQ and Δ total HOAs showed normal distribution, with *p*-values of 0.11 and 0.67, respectively. In the LASIK cohort, Δ total HOAs showed a Spearman’s correlation of 0.529 with Δ SEQ (*p* = <0.05, 95% CI: 0.033 to 0.056). For PRK, the correlation between Δ total HOAs and Δ SEQ was 0.373 (*p* < 0.05, 95% CI: 0.034 to 0.070). Similarly in the SMILE cohort, Δ total HOAs exhibited a Pearson’s correlation of 0.570 with Δ SEQ (*p* < 0.05, 95% CI: 0.033 to 0.085) (Figure 3).

**Figure 4:**
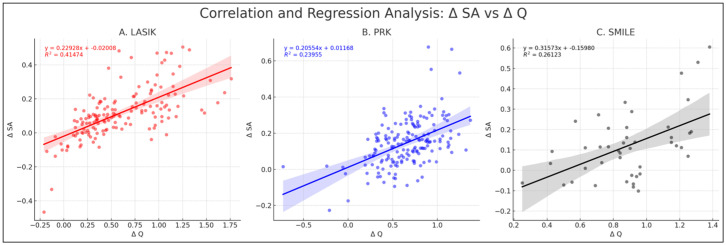
**Correlation and regression analysis of Δ SA vs. Δ Q.** This figure comprises three plots representing the correlation and regression analysis for the change in Δ SA against the change in Q-value (Δ Q) across LASIK (**A**), PRK (**B**), and SMILE (**C**). Each scatter plot with a linear regression line shows the equation of the regression line and the corresponding R^2^ value. PRK = photorefractive keratectomy, LASIK = laser-assisted in situ keratomileusis, SMILE = small incision lenticule extraction, HOAs = higher-order aberrations, SA = spherical aberration.

Across the three procedures, significant positive correlations were consistently observed between changes in SA and Q-value. Δ SA versus Δ Q in LASIK displayed the strongest Spearman’s correlation of 0.686 (*p* < 0.05, 95% CI: 0.592 to 0.762). In PRK, a moderate positive Spearman’s correlation of 0.4503 (*p* < 0.05, 95% CI: 0.323 to 0.562) was noted, while SMILE showed a relatively weaker yet significant correlation of 0.386 (*p* < 0.05, 95% CI: 0.101 to 0.613). A comparison between the correlations of LASIK and PRK indicated a significant difference in correlation strengths between Δ SA and Δ Q when comparing LASIK to both PRK (z = 3.17, *p* < 0.05) and SMILE (z = 2.46, *p* < 0.05). However, no significant difference was observed between PRK and SMILE (z = 0.446, *p* = 0.66) (Figure 4).

## 4. Discussion

Variations in pre-operative sphere, cylinder, and SEQ within the SMILE cohort can be attributed to the capability of SMILE to correct up to 10.0 D of myopia, compared to LASIK or PRK, which correct up to 8.0 D and 9.75 D, respectively [5,6]. While LASIK and PRK procedures can correct up to 6 D of astigmatism, SMILE can only correct up to 3 D [7], accounting for the lower average cylinder observed in pre-operative SMILE patients (Table 1). Similarly, PRK showed significant differences in pre-operative pachymetry, keratometry measurements, total HOAs, and vertical coma, as individuals with a pachymetry of <500 µm were excluded from LASIK and SMILE, leading to a selection of PRK for individuals with thinner corneas.

All procedures resulted in increased total HOAs, aligning with established outcomes for corneal refractive surgery due to corneal shape modification [8,9]. The increase seen in both post-LASIK and SMILE vertical coma may be linked to the inherent structural manipulation of the procedures, which could introduce more vertical asymmetry than PRK’s surface ablation technique [10]. Effect size analysis further revealed that SMILE, like its impact on total HOAs, had a more pronounced effect on vertical coma than LASIK and PRK. This finding is supported by Chen X et al. [10], who reported higher vertical coma after SMILE versus wavefront-guided femtosecond LASIK. These differences might be associated with SMILE’s unique centration technique, which employs the corneal vertex as a reference point, diverging from the pupillary center used in LASIK and PRK, aligning with findings from the existing literature [6,10,11,12,13,14]. In LASIK and PRK, variations in pupil size relative to treatment zones result in distinct HOA inductions. Further context to these findings is provided in Table A1, which outlines the rates of HOA induction found in previous studies.

The changes in SA and Q-value following LASIK, PRK, and SMILE could be explained by the central corneal flattening inherent in these procedures [15]. While this study observed variations within individual pre- and post-test groups, the overall lack of significant differences between the pre-surgical groups or the post-surgical groups in SA across all procedures may indicate a broadly similar impact on this type of aberration. Interestingly, an assessment of the variations in post- vs. pre-surgical change in SA among the procedures via effect size analysis revealed nuanced differences, particularly between LASIK and PRK. PRK showed a median absolute change in SA of 1.54 times greater compared to LASIK, suggesting that while the general trend in aberration increase is similar, the extent can vary depending on the specific procedure. Miraftab et al. [16] found no significant differences in spherical aberration changes between LASIK and PRK. However, studies by Jahadi Hosseini et al. [17] and Russo et al. [18] noted a greater induction of spherical aberration in LASIK compared to PRK. LASIK’s strong correlation of ΔSA/ΔQ suggests that altering the Q-value might have a greater impact on minimizing spherical aberration induction with LASIK compared to PRK or SMILE. In contrast, rates of change in SA for a given Q for SMILE and PRK were not significantly different, suggesting that there is likely no optimal procedure among these two which would induce SA at a lower rate for a given change in Q-value [19,20].

The PRK group also demonstrated a greater induction of oblique trefoil, further characterized by a rank-biserial correlation of −0.19 and a 12-fold difference in relation to LASIK. However, despite the statistical significance of PRK’s larger impact on oblique trefoil, the clinical implications are potentially limited due to the minor influence of oblique trefoil on overall retinal image quality [16]. Biscevic et al. [21] also found that PRK induced greater trefoil changes compared to LASIK. Nonetheless, the unique impact of PRK on vertical coma and oblique trefoil may highlight a specific interaction between the technique and the aberrations.

One of the main limitations of this study is the disparity in sample sizes between the SMILE cohort and the LASIK/PRK cohorts. However, SMILE is a newer procedure at our institution, and we do not believe the uneven group sizes affected the results related to higher-order aberrations, mirroring the current literature [14,16]. Additionally, it is important to emphasize that all of our patients were emmetropic, with satisfactory visual outcomes, and none of them required enhancement, reflecting that visual disturbances were due to HOAs not lower-order aberrations such as myopia or astigmatism. Some may argue that there is a selection bias, as the SMILE and PRK cohorts were not similar pre-operatively, but this is the apparent nature of patient selection, as those who typically undergo SMILE have a higher SEQ and sphere with a lower cylinder, while those who undergo PRK typically have thinner corneas [22]. These parameters were determined jointly by patients and clinicians rather than being randomly assigned. While it could be argued that one eye per patient should have been randomly selected rather than using both eyes, the GEE formula was employed to rectify inter-eye variability.

## 5. Conclusions

Each of the three procedures exhibited a significant rise in total HOAs, with a positive correlation observed between SEQ, Q-value, and the magnitude of induced HOAs. Vertical coma and spherical aberration emerged as the primary contributors to the overall increase in aberration magnitude, with SMILE showing the most pronounced effect, potentially attributed to its unique corneal apex fixation compared to the central axis alignment in LASIK and PRK. Notably, PRK displayed a noteworthy stability in vertical coma, contrasting with the other procedures, and demonstrated a distinct elevation in oblique trefoil, although the clinical implications of the latter remain uncertain.

## Figures and Tables

**Table 1:** **Pre-operative demographics across procedural groups**. D = diopter, PRK = photorefractive keratectomy, LASIK = laser-assisted in situ keratomileusis, SMILE = small incision lenticule extraction. Asterisks (*) denote statistical significance.

Demographic	LASIK	PRK	SMILE	*p*-Values
**Patients**	83	93	24	
**Eyes**	*n* = 154	*n* = 173	*n* = 44	
**Age Mean ± SD**	34.0 ± 7.2	33.5 ± 5.6	33.1 ± 6.0	0.6224
**Age Range**	20, 51	20, 44	22, 45	
**Male-to-Female Ratio**	49 to 34	43 to 50	11 to 13	0.199
**Sphere ± SE (D)**	−2.997 ± 0.220	−3.327 ± 0.168	−5.185 ± 0.350 *	<0.001
**Sphere Range (D)**	−7.5, 1.5	−7.75, 1	−8.5, −2.25	
**Cylinder ± SE (D)**	−1.024 ± 0.1	−0.964 ± 0.09	−0.443 ± 0.072 *	0.011
**Cylinder Range (D)**	−5.75, 0	−4.25, 0	−1.25, 0	
**Spherical Equivalent (D)**	−3.509 ± 0.218	−3.809 ± 0.162	−5.406 ± 0.346 *	<0.001
**Spherical Equivalent Range (D)**	−8.375, −0.25	−8, −0.375	−8.5, −2.75	
**Pachymetery Min (µm)**	548.896 ± 2.719	522.664 ± 3.217 *	551.864 ± 6.832	<0.001
**Pachymetery Apex (µm)**	552.734 ± 2.694	526.850 ± 3.179 *	556.023 ± 0.023	<0.001
**Pachymetery Pupil (µm)**	551.994 ± 2.701	526.26 ± 3.185 *	555.568 ± 6.681	<0.001
**Keratometry Mean ± SE (D)**	43.726 ± 0.146	44.175 ± 0.129	43.814 ± 0.212	0.08
**Keratometry Range (D)**	39.95, 47.2	41.2, 47.85	41.7, 46	
**Total HOAs (µm)**	0.348 ± 0.008	0.43 ± 0.012 *	0.329 ± 0.011	<0.001
**Total HOAs Range (µm)**	0.173, 0.625	0.197, 0.837	0.185, 0.504	
**Spherical Aberration (µm)**	0.197 ± 0.009	0.178 ± 0.009	0.183 ± 0.011	0.298
**Spherical Aberration Range (µm)**	−0.065, 0.434	−0.077, 0.417	−0.016, 0.309	
**Vertical Coma (µm)**	−0.092 ± 0.014	−0.197 ± 0.021 *	−0.1 ± 0.024	<0.001
**Vertical Coma Range (µm)**	−0.349, 0.289	−0.656, 0.711	−0.345, 0.18	
**Horizontal Coma (µm)**	0.027 ± 0.006	0.036 ± 0.006	0.043 ± 0.009	0.361
**Horizontal Coma Range (µm)**	−0.507, 0.442	−0.427, 0.422	−0.242, 0.249	
**Oblique Trefoil (µm)**	−0.029 ± 0.008	−0.01 ± 0.01	−0.014 ± 0.013	0.314
**Oblique Trefoil Range (µm)**	−0.233, 0.187	−0.351, 0.268	−0.184, 0.199	
**Horizontal Trefoil (µm)**	−0.004 ± 0.005	−0.006 ± 0.007	−0.011 ± 0.011	0.872
**Horizontal Trefoil Range (µm)**	−0.266, 0.214	−0.294, 0.222	−0.175, 0.201	

**Table 2:** **Mean values of total HOAs and subtypes pre- and post-procedure**. This table presents the mean values of total HOAs and their five subtypes pre- and post-LASIK, PRK, and SMILE procedures. The table also includes standard error values for both pre-test and post-test measurements, along with *p*-values to assess the significance of changes between pre- and post-procedure values. PRK = photorefractive keratectomy, LASIK = laser-assisted in situ keratomileusis, SMILE = small incision lenticule extraction, HOAs = higher-order aberrations.

HOA Subtype Name	Procedure Name	Mean Pre-Test (μm)	Mean Post-Test (μm)	*p*-Value
Total HOAs	LASIK	0.348 ± 0.00654	0.555 ± 0.0145	<0.05
	PRK	0.431 ± 0.0984	0.599 ± 0.0150	<0.05
	SMILE	0.329 ± 0.00947	0.617 ± 0.0223	<0.05
Spherical Aberration	LASIK	0.197 ± 0.00656	0.313 ± 0.0117	<0.05
	PRK	0.178 ± 0.00709	0.324 ± 0.0105	<0.05
	SMILE	0.183 ± 0.0950	0.305 ± 0.0254	<0.05
Vertical Coma	LASIK	−0.0922 ± 0.0106	−0.127 ± 0.0192	<0.05
	PRK	−0.197 ± 0.0168	−0.192 ± 0.0198	0.78
	SMILE	−0.0990 ± 0.0184	−0.323 ± 0.0312	<0.05
Horizontal Coma	LASIK	0.0267 ± 0.0106	0.0314 ± 0.0193	0.60
	PRK	0.0362 ± 0.0112	0.0472 ± 0.0182	0.45
	SMILE	0.0426 ± 0.0156	0.00718 ± 0.0285	0.22
Oblique Trefoil	LASIK	−0.0290 ± 0.00746	−0.0156 ± 0.0103	0.18
	PRK	−0.00953 ± 0.00843	−0.0413 ± 0.0108	<0.05
	SMILE	−0.0145 ± 0.0121	−0.0134 ± 0.0182	0.94
Horizontal Trefoil	LASIK	−0.00395 ± 0.00649	−0.00208 ± 0.00912	0.84
	PRK	−0.00574 ± 0.00730	0.000162 ± 0.00858	0.22
	SMILE	−0.0110 ± 0.0137	0.0239 ± 0.0181	0.08

**Figure A2:**
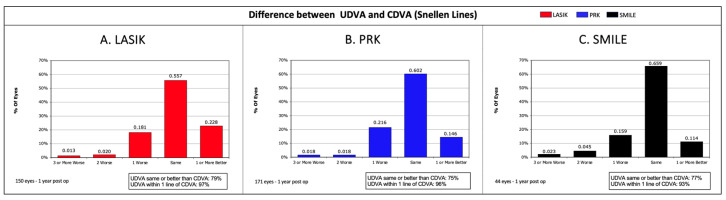
One-year post-operative differences in Snellen lines between UDVA and CDVA by surgery type (LASIK ((**A**); red), PRK ((**B**); blue), and SMILE ((**C**); black)).

**Figure A4:**
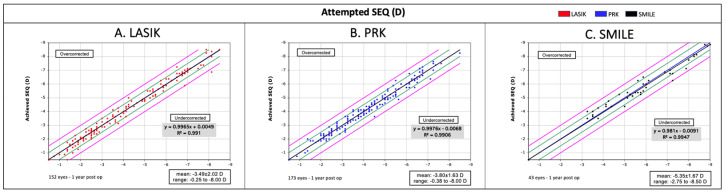
One-year post-operative achieved SEQ change vs. attempted SEQ change by surgery type (LASIK ((**A**); red), PRK ((**B**); blue), and SMILE ((**C**); black)). One-year post-operative achieved SEQ change vs. attempted SEQ change by surgery type. Blue line indicates at-tempted = achieved, green lines indicate +/− 1.00 D, magenta lines indicate +/− 1.50 D.

**Figure A5:**
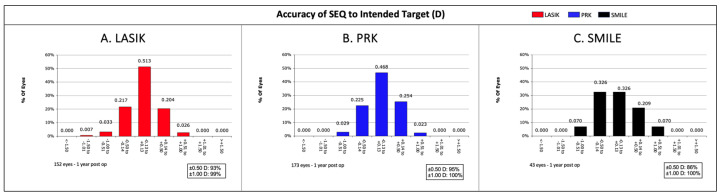
One-year post-operative accuracy of SEQ to intended target by surgery type (LASIK ((**A**); red), PRK ((**B**); blue), and SMILE ((**C**); black)).

**Figure A6:**
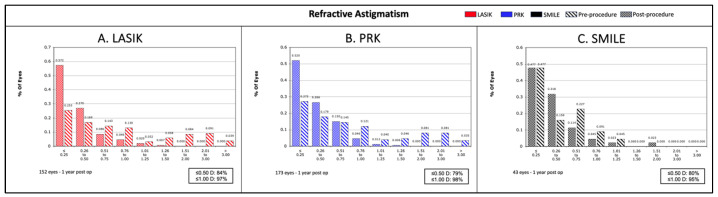
One-year post-operative refractive astigmatism by surgery type (LASIK ((**A**); red), PRK ((**B**); blue), and SMILE ((**C**); black)).

**Figure A7:**
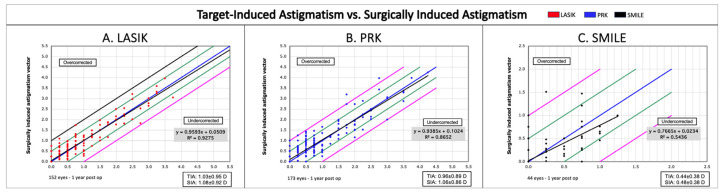
One-year post-operative targeted astigmatism vs. surgically induced astigmatism by surgery type (LASIK ((**A**); red), PRK ((**B**); blue), and SMILE ((**C**); black)). One-year post-operative targeted astigmatism vs. surgically induced astigmatism by surgery type. The blue line indicates attempted = achieved, green lines indicate +/− 0.50 D, magenta lines indicate +/− 1.00 D.

**Figure A8:**
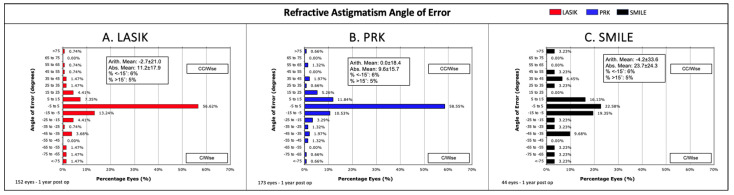
One-year post-operative refractive astigmatism angle of error by surgery type (LASIK ((**A**); red), PRK ((**B**); blue), and SMILE ((**C**); black)).

**Figure A9:**
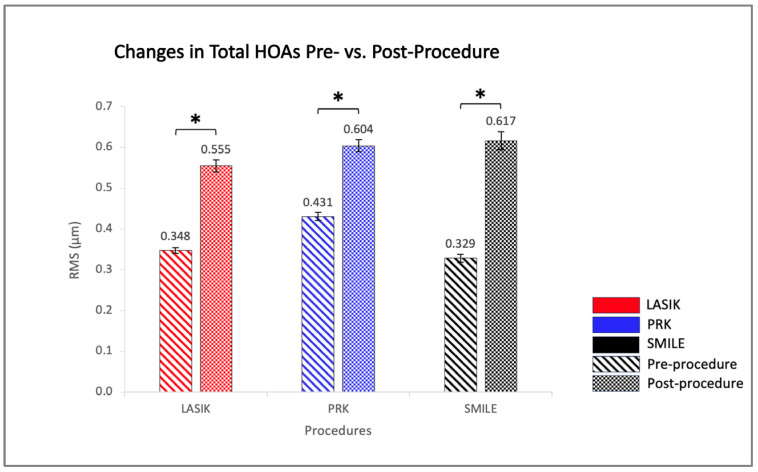
**Comparison of total HOAs pre- and post-operatively**. This figure visualizes the changes in total HOAs for each surgical procedure, represented by bars (pre-operative: diagonal stripe patterns, post-operative: solid colors). Red is used for LASIK, blue for PRK, and black for SMILE. Error bars indicate standard error, and asterisks (*) denote statistically significant differences (*p* < 0.05). PRK = photorefractive keratectomy, LASIK = laser-assisted in situ keratomileusis, SMILE = small incision lenticule extraction, HOAs = higher-order aberrations.

**Figure A10:**
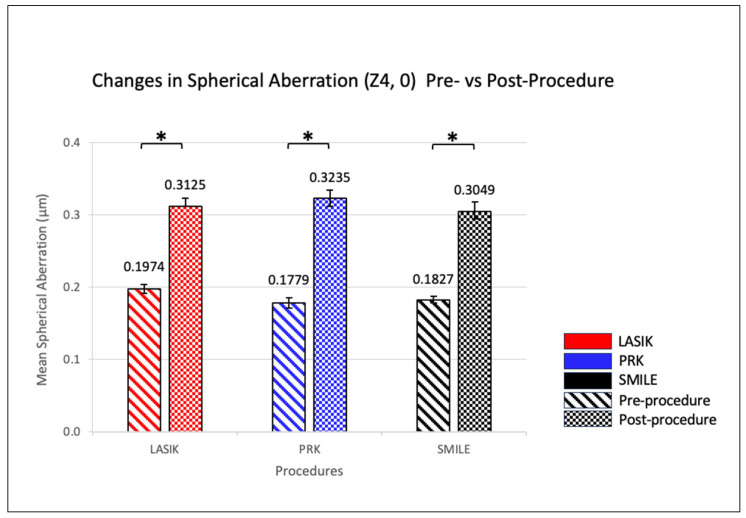
**Comparison of spherical aberration (Z4, 0) pre- and post-operatively**. This figure illustrates the changes in spherical aberration, with pre-operative measures in diagonal stripe patterns and post-operative in solid colors. Red indicates LASIK, blue PRK, and black SMILE. Error bars represent standard error, and asterisks (*) signify statistically significant differences (*p* < 0.05). PRK = photorefractive keratectomy, LASIK = laser-assisted in situ keratomileusis, SMILE = small incision lenticule extraction, HOAs = higher-order aberrations.

**Figure A11:**
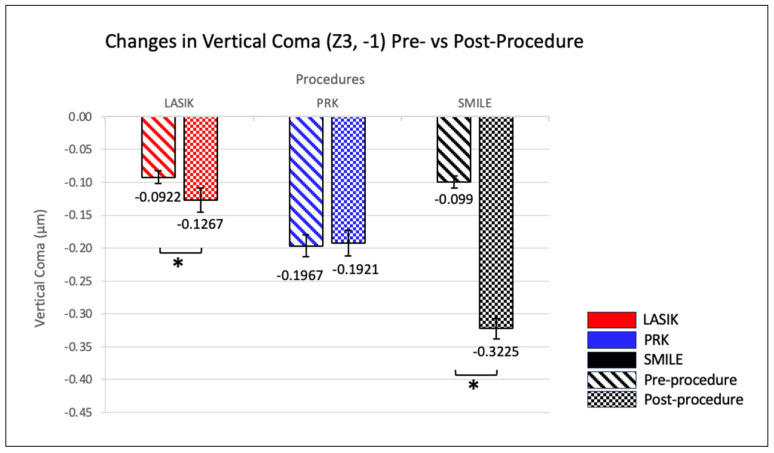
**Comparison of vertical coma (Z3, −1) pre- and post-operatively**. This figure showcases the changes in vertical coma for each procedure, with pre-operative values in diagonal stripes and post-operative in solid colors. Red denotes LASIK, blue PRK, and black SMILE. Error bars denote standard error, and asterisks (*) highlight statistically significant differences (*p* < 0.05). PRK = photorefractive keratectomy, LASIK = laser-assisted in situ keratomileusis, SMILE = small incision lenticule extraction, HOAs = higher-order aberrations.

**Figure A12:**
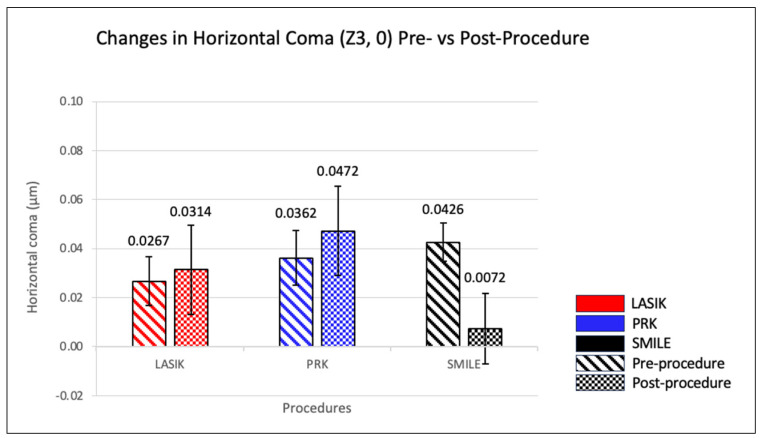
**Comparison in horizontal coma (Z3, 0) pre- and post-operatively**. This figure presents the changes in horizontal coma, with pre-operative bars in diagonal stripes and post-operative in solid colors. Colors represent different procedures: red for LASIK, blue for PRK, and black for SMILE. Error bars indicate standard error. PRK = photorefractive keratectomy, LASIK = laser-assisted in situ keratomileusis, SMILE = small incision lenticule extraction, HOAs = higher-order aberrations.

**Figure A13:**
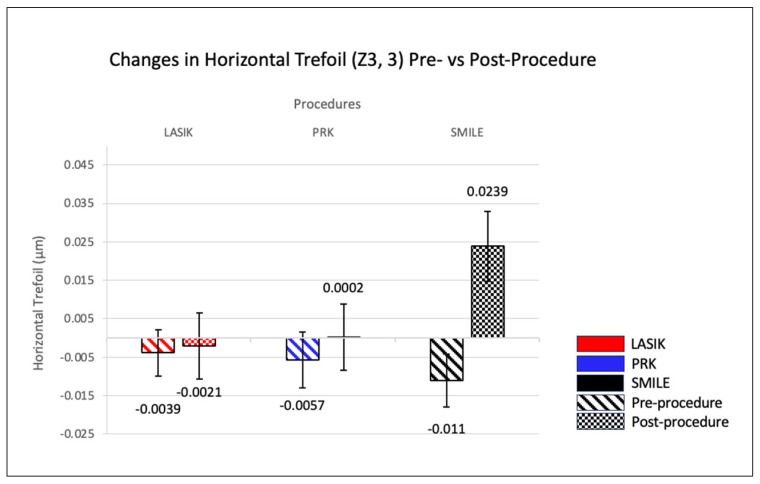
**Comparison of horizontal trefoil (Z3, 3) pre- and post-operatively**. This figure depicts the changes in horizontal trefoil for each procedure, with pre-operative values in diagonal stripes and post-operative in solid colors. Red signifies LASIK, blue PRK, and black SMILE. Error bars denote standard error. PRK = photorefractive keratectomy, LASIK = laser-assisted in situ keratomileusis, SMILE = small incision lenticule extraction, HOAs = higher-order aberrations.

**Figure A14:**
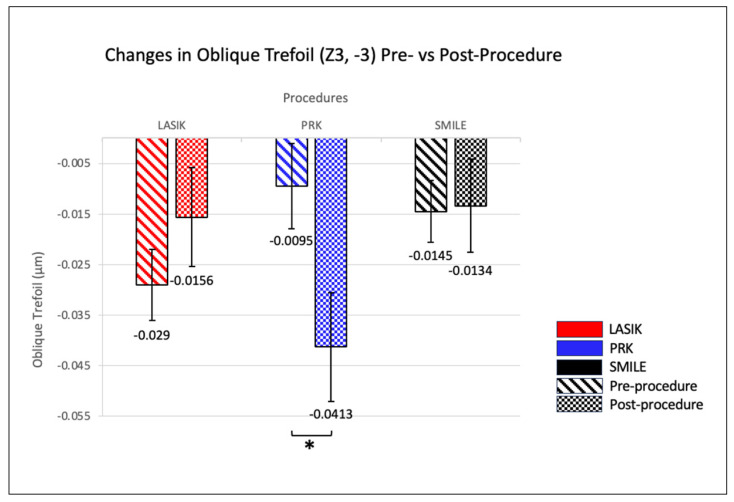
**Comparison of oblique trefoil (Z3, −3) pre- and post-operatively**. This figure displays the changes in oblique trefoil, with pre-operative values in diagonal stripes and post-operative in solid colors. The color scheme includes red for LASIK, blue for PRK, and black for SMILE. Error bars represent standard error, and asterisks (*) indicate statistically significant changes (*p* < 0.05). PRK = photorefractive keratectomy, LASIK = laser-assisted in situ keratomileusis, SMILE = small incision lenticule extraction, HOAs = higher-order aberrations.

## Data Availability

All data analyzed during this study are included in this published article as citations in the results section.

## Appendix A

**Table A1:** **Comparative analysis of HOA induction after refractive procedures.** This table presents a comprehensive comparison of the induction of higher-order aberrations (HOAs) based on our study findings and other published reports. The included studies utilized diverse imaging modalities to evaluate the impact of different refractive procedures on ocular aberrations. Variations in the imaging modalities, number of eyes examined, procedures performed, and follow-up duration are recorded across the studies. Results that were not statistically significant are denoted as “NS”, while “NR” indicates data that were not reported in the original publication. PRK = photorefractive keratectomy, LASIK = laser-assisted in situ keratomileusis, SMILE = small incision lenticule extraction, HOAs = higher-order aberrations.

Study	Imaging Modality	Eyes (Approx. Eyes/Patient)	Procedures	Comments	Total HOAs (μm)	Spherical Aberration (μm)	Vertical Coma (μm)	Horizontal Coma (μm)	Oblique Trefoil	Horizontal Trefoil
Our Study	Pentacam	154 (2)	LASIK	1 year	0.2070 ± 0.1974	0.1160 ± 0.1665	−0.0348 ± 0.2722	NS	NS	NS
		173 (2)	PRK		0.1680 ± 1.3092	0.1460 ± 0.1666	NS	NS	−0.0318 ± 0.1802	NS
		44 (2)	SMILE		0.2880 ± 0.1607	0.1220 ± 0.6523	−0.2240 ± 0.2403	NS	NS	NS
Wu, et al., 2021 [15]	Pentacam	88 (2)	LASIK	6 months	0.31 ± 0.25	0.17 ± 0.11	0.24 ± 0.18	0.14 ± 0.16	NR	NR
		64 (2)	PRK		0.31 ± 0.18	0.2 ± 0.15	0.11 ± 0.15	0.12 ± 0.18		
Kang E., et al., 2022 [22]	Pentacam	45	LASIK	6 months	NS	NS	NS		NS	
		45	SMILE		0.151 ± 0.178	0.074 ± 0.162	0.181 ± 0.233		NS	
Miraftab M., et al., 2021 [16]	Sirius	124 (1)	LASIK	Stratified by moderate or high myopic patients; moderate results have been included here; 12-month data; 6 mm pupil	0.38 ± 0.36	0.24 ± 0.1	0.25 ± 0.23		NS	
		124 (1)	PRK		0.33 ± 0.25	0.26 ± 0.13	0.24 ± 0.24		NS	
		124 (1)	SMILE		0.16 ± 0.32	0.11 ± 0.09	0.16 ± 0.23		NS	
